# Beyond the Bench: A Two-Way Street: Building Lasting Community Connections

**DOI:** 10.1289/ehp.114-a581

**Published:** 2006-10

**Authors:** Tanya Tillett

It is human nature to remain committed to endeavors in which one feels personally invested. For the parents and children involved in studies at the Mount Sinai Center for Children’s Environmental Health and Disease Prevention Research, most of whom are from low-income, minority communities such as East Harlem and the Bronx, this sense of commitment plays an important role in their continued participation in such studies. The center’s Community Outreach and Translation Core (COTC) encourages this community kinship by partnering with community organizations to create workshops and educational activities that help keep children and their parents engaged in the studies.

According to COTC director Luz Claudio, the COTC staff have designed activities that pick up where organized educational activities at school leave off, encouraging children to learn new, useful information they can share with their parents. The activities are also culturally relevant and easy to take advantage, which makes it easy to keep them going.

Claudio says one main goal of these programs is to expose the study participants to realistic, positive role models in the medical profession to encourage their interest in future medical careers. Another is to remind study participants that, through their participation in center studies, they are part of a national effort to improve and protect children’s health. “The COTC educational activities provide direct benefits to the participants that go beyond their participation as study subjects providing data,” says Claudio. “They are truly our partners in the scientific endeavor.”

In one current collaborative project popular with kids and parents alike, COTC staff have joined with the nonprofit City Parks Foundation to produce educational workshops aimed at increasing study participants’ physical activity. Claudio points to a study published in the September 2006 issue of the *American Journal of Public Health* showing that low-income and minority neighborhoods have far fewer commercial physical activity–related facilities available. “This makes our workshops all the more important for these communities because there are few gyms or other sports facilities that are accessible to them,” she says.

The workshops offered through the City Parks Foundation collaboration introduce the wonders of the outdoors to children who might not have spent much time there. Workshops include “Trees, Leaves and Worms” (observing nature in action), “My Nature Journal” (recording those observations), and ice-skating excursions in Central Park. “We want parents to learn that they can use New York City parks as learning resources that also provide free health benefits. Parents who become excited by the experiences are more likely to integrate these types of excursions into their children’s lives,” says Claudia DeMegret, director of education at the City Parks Foundation.

Other collaborations include family mini-golf with the Randall’s Island Sports Foundation, a “Mad Hot Dancing!” class with salsa dancer Rodney Lopez (featured in the movie *Mad Hot Ballroom*), and a “Yummy Good” cooking class in partnership with the community organization Little Sisters of the Assumption. The center also distributes regular newsletters and fact sheets telling families where the program’s outdoor activities are conducted.

Other workshops are designed specifically to demystify the scientific process and reinforce to the study participants how integral they are to the program. Kids can look at their own cells under a microscope in the “Your Body, Your Cells” workshop. They and their parents also learn to distinguish reliable and unreliable sources of health information on the web in the “On-line for Health” workshop. Scavenger hunts afford the children the opportunity to learn about different kinds of plastics and their varying levels of safety (“Plastics and More Plastics”), and they are also introduced to genetics and heredity (“Do These Genes Make Me Look Fat, and Other Things Genes Do”).

Parents appreciate the diversity of entertaining learning opportunities offered to the children through the programs, and note how the kids connect these experiences to their role in the center’s research projects. One parent (who is unidentified to protect the privacy of the study participant) observes, “They enjoy playing in the grass, being in the dirt, collecting leaves. . . . [After an event] they remember and talk about some of the things they did. I think the more they’re exposed to things, the more interested they become in the study.”

## Figures and Tables

**Figure:**
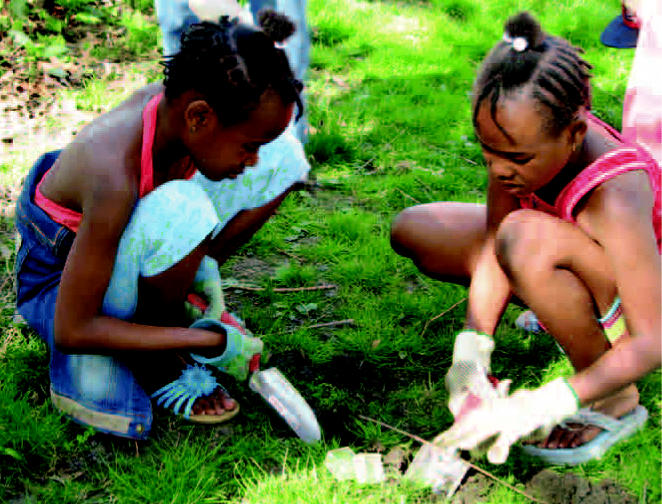
Getting down and dirty with the environment A program of the Mount Sinai Center for Children’s Environmental Health and Disease Prevention Research and the New York City Parks Foundation engages children in activities that teach them the relevance of the environment—and environmental research—to their health.

